# Adherence to antiretroviral treatment and associated factors among people living with HIV and AIDS in CHITWAN, Nepal

**DOI:** 10.1186/s12889-019-7051-3

**Published:** 2019-06-10

**Authors:** Sujan Neupane, Govinda Prasad Dhungana, Harish Chandra Ghimire

**Affiliations:** 1 Chitwan Sakriya Women’s foundation, Bharatpur, Chitwan Nepal; 2 Department of Statistics, Birendra Multiple Campus, Bharatpur, Nepal; 3 Tribhuvan University, Kirtipur, Nepal

**Keywords:** ART, Adherence, PLHIV, Nepal, Side effects

## Abstract

**Background:**

Adherence to ART is the primary determinant of viral suppression and the risk of transmission, disease progression and death. Adherence of at least 95% is needed for optimal suppression. This study aimed at determining the adherence to Anti-Retroviral Therapy (ART) and its associated factors among People Living with HIV and AIDS in ART Center of Chitwan, Nepal.

**Methods:**

A descriptive cross-sectional study was conducted among 231 clients aged 18 years to 49 years taking ART from Bharatpur Hospital of Chitwan and those who have been enrolled in ART for at least 6 months, were interviewed. Systematic Sampling technique was used. Semi-structured questionnaire was prepared by taking reference from the AIDS Clinical Trial group questionnaire (ACTG). Adherence was measured by patient self report. Data was entered Epi Data 3.1 and analyzed using Statistical Package for Social Sciences (SPSS) software where the *P* value of < 0.05 was accepted as being statistically significant. The independent variables which were found significant at *p*-value 0.10 in bivariate analysis were fitted in multivariable logistic regression model. Multivariable logistic regression model was performed to know the net effect of the independent variables on Adherence to ART medication.

**Results:**

The overall adherence in the last month was found to be 87.4%. Wrist watch and mobiles were seen as a facilitating factor for taking ART on time as clients taking ART used to set alarm to get informed of the medication time. Adherence was associated with female sex (AOR = 10.550 CI: 1.854–60.046), family consisting only parents and their children (AOR = 4.877, CI: 1.246–19.079), having no habit of taking alcohol (AOR = 5.842 CI: 1.294–26.383), HIV duration of more than 3 years (AOR = 10.055 CI: 2.383–42.430), picking up ART medications on their own (AOR = 7.861, CI: 1.670–36.998) and not having side effects of ART (AOR = 8.832, CI: 2.059–37.890).

**Conclusion:**

Identifying and evaluating the problems faced by ARV drug users can foster the achievement of ART related goals and addressing ART related problems in a rational way. Effective and appropriate monitoring of non adherence behaviors can help patients increase adherence level fostering improvement in treatment outcome.

**Electronic supplementary material:**

The online version of this article (10.1186/s12889-019-7051-3) contains supplementary material, which is available to authorized users.

## Background

An estimated 36.7 million people are currently living with HIV and AIDS and 1.8 new infections occur each year. Approximately a Million (830000–1.2 million) people died from AIDS related illness [1]. As of 2016, national estimates indicate that approximately 39,397 people are living with HIV [2]. A national program providing free access to Anti Retroviral Therapy (ART) began in Nepal during 2004 [3]. At the end of July 2016 a total of 16,499 clients have been enrolled into treatment from 65 sites in 59 districts [4]. The retention of people on antiretroviral therapy in 2015 was 83.7% after 12 months and 78% after 24 months [5]. National HIV Strategic plan 2016–21 aims to achieve 90% of HIV infected children and adults will be receiving ART and viral Suppression to be achieved to 90% [5].

Target 3.3 of the sustainable development goals (SDG) aims to end the AIDS Epidemic by 2030 [6]. With its “treat-all” recommendation, WHO removes all limitations on eligibility for ART among people living with HIV; all populations and age groups are now eligible for treatment, including pregnant women and children [7]. Only 40% of the people living with HIV are receiving ART and only 36% of those being treated have suppressed viral load which implies that the adherence rate in Nepal is quite low [8]. This situation suggests that Nepal is far away from achieving the 90–90-90 target which implies that by 2020: 90% of all people living with HIV will know their HIV status, 90% of all people with diagnosed HIV infection will receive sustained antiretroviral therapy, and 90% of all people receiving antiretroviral therapy will have viral suppression [6]_._

A mixed method study carried out in Nepal by Wasti et al. in 2009 reported adherence rate of 86% [9]. Similarly a study carried out in Kathmandu valley by Shigdel et al. in 2012 reported adherence rate of 86.7% [10]. A cross sectional study which was carried out by Bam et al. in Nepal in 2015 revealed the adherence rate of 94.8% [11]

Adherence is the extent to which a person’s behavior – taking medication, following a diet and/or changing lifestyle – corresponds with agreed recommendations from a health worker. Adherence to ART may also be challenging in the absence of supportive environments for people living with HIV and in the presence of HIV-related stigma and discrimination. Medication-related factors may include adverse effects and the complexity of dosing regimens. Health system factors include distance to health services, long waiting times to receive care, receiving only 1 month’s supply of drugs, pharmacy stock-outs and the burden of direct and indirect costs of care [7]. Poor adherence can lead to the virological failure of cheap first-line treatment regimens and the spread of multi-drug resistant forms of the virus, resulting in a public health calamity [9].

The key to the success of ART programs and prevention of treatment failures is hinged on consistently high adherence levels. Scaling up of ARVs alone is definitely not the answer when adherence inconsistencies are not tackled. Therefore, the first step to solving this problem is to assess the determinants of adherence to ART [12]. The risk of transmission of resistant viruses and limited future treatment options due to poor adherence makes adherence a public health concern [13]. The study was initiated with the aim of determining the adherence to Anti-Retroviral Therapy (ART) and its associated factors among People living with HIV and AIDS in ART Center of Chitwan, Nepal.

## Methods

### Study design and population

This was a cross sectional study carried out from 29th August to 24th September 2017 in ART center of Bharatpur hospital of Chitwan District. The study populations were sexually active clients of age 18 years to 49 on ART Center of Bharatpur Hospital.

### Sample size

The sample was determined by.

$$ {\mathrm{n}}_{=}\ {\frac{N{\mathrm{Z}}_{\upalpha}^2 pq}{d^2\left(N-1\right)+{\mathrm{Z}}_{\upalpha}^2 pq}}^{35}. $$

z_α_ = 1.96 for 95% confidence interval.

p = Prevalence of ART Adherence.

(*p* = 0.87) [14], q = 1-p = (1–0.87)

d = precision or error in the study = 0.03.

Total eligible study population were (N) =370.

Sample size =210 + 10% non response rate.

= 231.

### Sampling method

Systematic sampling was done for the study. First of all the list of clients was obtained on excel sheet from the ART Center of Chitwan. A total of 370 Clients of age 18 and above and 49 were actively enrolled in ART. After the eligible respondent list was obtained sample size was calculated and to select the respondents, each alternate sample present at the day of data collection was taken for the study. Adherence was calculated by using the formula from National ART Guideline, 2014.

$$ \mathrm{Adherence}\ \mathrm{percentage}=\frac{\mathrm{Number}\ \mathrm{of}\ \mathrm{pills}\ \mathrm{taken}\ \mathrm{during}\ \mathrm{the}\ \mathrm{specific}\ \mathrm{period}\left(1\ \mathrm{month}\right)}{\mathrm{Number}\ \mathrm{of}\ \mathrm{pills}\ \mathrm{to}\ \mathrm{be}\ \mathrm{taken}\ \mathrm{during}\ \mathrm{that}\ \mathrm{specific}\ \mathrm{period}\ \left(1\ \mathrm{month}\right)}\ast 100 $$

The Adherence performance Chart was used to classify optimal and suboptimal adherence which has been presented in Table 1.

**Table 1:** Adherence Performance Chart

No of pills per day		Percentage of Adherence		
		> 95%	80–95%	< 80%
1	Number of pills missed in a month	1	2 to 6	7 or more
2		3 or less	4 to 12	13 or more
3		4 or less	5 to 18	19 or more
4		6 or less	7 to 24	25 or more

### Data collection technique and tools

Semi-structured questionnaire was administered for face to face interview for self reported adherence. In this study, for adherence assessment, last 1 month self-reported adherence as mentioned in National Consolidated Guidelines for Treating and Preventing HIV in Nepal, 2014 was adopted. Semi-structured questionnaire was prepared by taking reference from the AIDS Clinical Trial group questionnaire (ACTG) and study conducted by WHO in 2006 to understand access to adherence. Questionnaire was first developed in English and translated into Nepali. Pretesting was done on 10% sample size in ART centre of Butwal to ensure the reliability.

The questionnaire tool has been attached within the Additional file 1 within this manuscript.

### Data management and analysis

The collected data was manually edited, coded and entered in database software Epi Data version 3.1. After that data was exported to Statistical Package for Social Science (SPSS) software version 21 for further statistical analysis. In Bi-variate analysis Chi-square test and odds ratio were used to test the significance of association between independent and dependent variables. The independent variables which were found significant at *p*-value 0.10 in bivariate analysis were fitted in multivariable logistic regression model. Multivariable logistic regression model was performed to know the net effect of the independent variables on Adherence to ART medication. There were total of 231 cases in analysis. Due to presence of outliers, 9 cases were excluded from analysis out of total cases in order to fit the model adequacy. The goodness of fit for the model was assessed by using Hosmer and Lemshow test which showed the model was statistically insignificant. Model adequacy was performed through Scatter plot of Standarized residual, Leverage value and Analog of Cooks influence.

## Results

### Adherence rate

Out of 231 respondents, 87.4% (95% CI: 83.2–91.6%) of them had an optimal adherence level and 12.6% of the respondents had an adherence level less than the optimal within the last month.. i.e. 87.4% of the respondents’ attained 95% adherence to prescribed ART regimen.

**Fig. 1:**
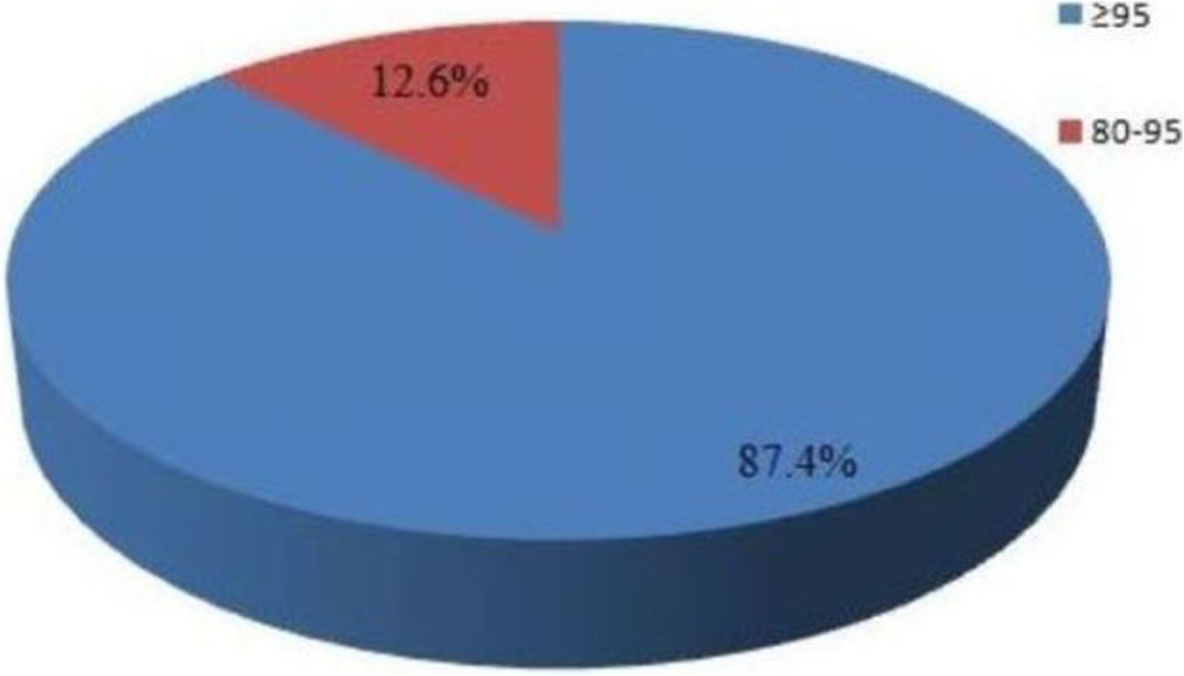
Adherence rate

Diagrammatic representation of the Adherence rate has been shown in Fig. 1.

### Descriptive analysis

The overall mean age of the respondents was 38.6. Majority (79.7%) of the respondents followed Hindu Religion. Nearly a half (50.6%) had primary level education. More than 3/5th (65.8%) of the respondents were married and a little above a quarter (27%) were widowed. More than a half (56.3%) was from nuclear family. Most of the respondents haven’t had alcohol (81.4%) or smoking habit (90%). Majority of the respondents had been infected with HIV (79.7%) and had been enrolled in ART (73.2%) for more than 3 years.

Results of descriptive analysis have been presented in Table 2.

**Table 2:** Baseline Characteristics

Characteristics	Number	Percent
Sex		
Female	134	58.0
Male	97	42.0
Age group		
18–24	9	3.9
25–34	50	21.6
35–44	124	53.7
> 44	48	20.8
Mean ± SD =38.55 ± 6.84		
Religion		
Hindu	184	79.7
Boudhha	23	10.0
Christian	22	9.5
Islam	2	0.9
Educational Status		
Illiterate	77	33.4
Primary ^B^	117	50.6
Secondary ^C^	37	16.0
Marital status		
Married	152	65.8
Widowed	62	26.8
Unmarried	15	6.5
Divorced/ Separated	2	0.9
Family type		
Nuclear	130	56.3
Joint	101	43.7
Disclosure status (*n* = 231)		
Yes	185	80.1
No	46	19.9
Fear of stigma		
No	122	52.8
Yes	109	47.2
Alcohol habit		
Yes^A^	43	18.6
No	188	81.4
Smoking habit		
Yes^B^	23	10.0
No	208	90.0
Duration of being HIV infected		
< 1 year	29	12.6
1–3 year	18	7.8
> 3 years	184	79.7
Duration of being enrolled in ART		
< 1 year	43	18.6
1–3 year	19	8.2
> 3 years	16	73.2
Side Effects		
No	184	79.7
Yes	47	20.3

Educational status: ^B^ includes Primary, lower secondary ^C^ includes secondary, higher secondary Alcohol habit: A includes Once a month, 2–3 times a month, Once or twice a week, 3–4 times a week, Nearly every day, daily. Smoking habit: B includes Never, 1–2 smoke per day, 3–4 smoke per day, 5–6 smoke per day, more than 6 smokes per day

### Multivariable logistic regression model of factors associated with adherence to ART

Female sex was found to be significantly associated with ART adherence in the multivariable logistic regression model by controlling for the other variables. Female were 11 times more likely to adhere to HIV medication compared to male (AOR = 10.550 CI: 1.854–60.046). The odds of ART adherence were 5 times higher in respondents from family consisting only parents and their children than those from family consisting more than parents and children (OR = 4.877, CI: 1.246–19.079). Those who do not drink alcohol are 6 times more likely to adhere to the HIV medication than those who do drink (OR = 5.842 CI: 1.294–26.383). Duration of HIV infection was found to be associated with improved adherence. Respondents who reported HIV duration of more than 3 years were 10 times more likely to have optimal adherence compared to those whose HIV infection duration is less than 3 years (AOR = 10.055 CI:2.383–42.430). Side effect was seen as one of the important factor influencing ART adherence. Those respondents who do not experience side effects were 9 times more likely to have optimal adherence compared to those who experience side effects from medication (OR = 8.832, CI: 2.059–37.890). Those who pick ART medicine on their own were found to be more adherent than those who don’t pick up ART medication on their own. Those who comes to receive ART medicine themselves in the ART center were 8 times more adherent compared to those who don’t receive ART medicine from the ART Center themselves (OR = 7.861, CI: 1.670–36.998).

The results of Multivariable logistic regression model of factors associated with adherence to ART have been presented in Table 3.

**Table 3:** Multivariable logistic regression model of factors associated with Adherence to ART

Characteristics	Unadjusted OR	Adjusted OR	*p* Value
Sex			
Female	2.548 (1.143–5.679)	10.550 (1.854–60.046)	0.008
Male	1	1	
Marital Status			
Widowed	3.687 (1.068–12.733)	2.989 (0.191–46.656)	0.435
Others	1.406 (0.302–6.550)	1.301 (0.196–8.644)	0.785
Married	1		
Family type			
Nuclear	1.990 (0.903–4.986)	4.877 (1.246–19.079)	0.023
Joint	1	1	
Alcohol Habit			
No	2.695 (1.150–6.317)	5.842 (1.294–26.383)	0.022
Yes	1	1	
Smoking habit			
No	3.699 (1.372–9.975)	0.318 (0.052–1.922)	0.212
Yes	1	1	
HIV Duration			
Less than 3 year	1	1	0.002
More than 3 year	2.347 (1.009–5.462)	10.055 (2.383–42.430)	
Perception on own health			
Better	2.597 (0.867–7.785)	4.204 (0.752–23.493)	0.102
Not better	1	1	
Side effects			
No	4.015 (1.769–9.110)	8.832 (2.059–37.890)	0.003
Yes	1	1	
Receiving ART Medicine own self			
No	1	1	
Yes	2.548 (1.143–5.679)	7.861 (1.670–36.998)	0.009
Client satisfaction			
Excellent	4.914 (0.785–30.747)	0.632 (0.040–10.062)	0.745
Poor/Good /Fair	1	1	

### Model adequacy test

In order to examine fitness of model with the observed data, several standard measures of model adequacy tests have been used. Log likelihood (LL) was used to access the overall fitness of the model. To see the degree of explanation by the covariates used in the fitted model on variation in level of adherence, pseudo R^2^ was calculated. Negelkerke R^2^ (pseudo R^2^) measure the proportion of the variation in the dependent variables that can be explained by predictors in the model. Here R_x_^2^ = 0.501which indicates that 50.1% of the variation in Adherence rate has been explained by the independent variables.

Results of Model Adequacy test have been presented in Table 4.

**Table 4:** Model Adequacy Test

Model Summary		
-2 Log likelihood	Cox & Snell R Square	Nagelkerke R Square
77.110	0.228	0.501
Hosmer and lemeshow		
Chi-square	Df	P value
1.817	8	0.986

### Scatter plot for outliers (standardized residual)

**Fig. 2:**
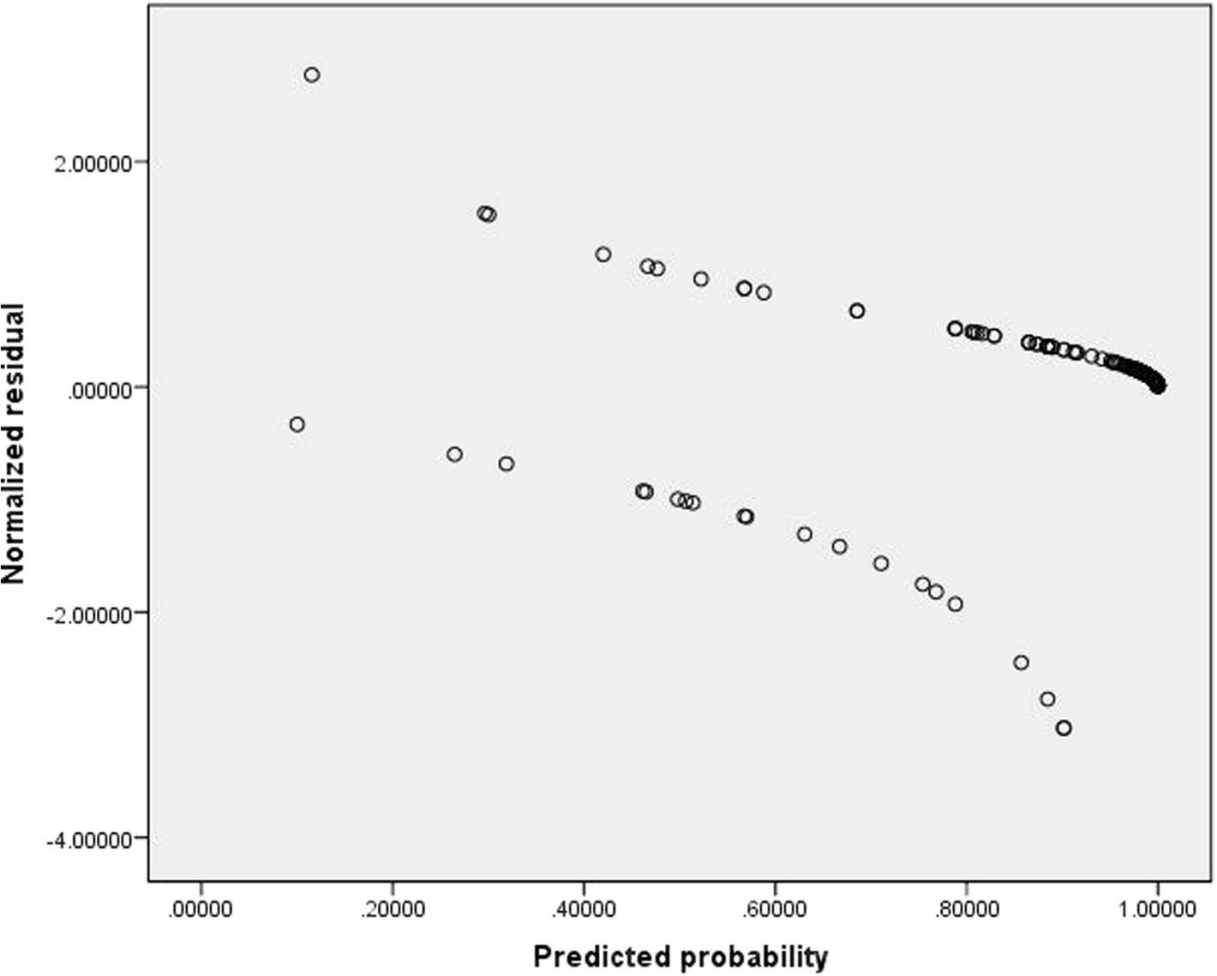
Scatter plots for outliers (Standardized residual)

Residual analysis was carried out via graphs. A check of the standardized residuals for the level of adherence is presented in the Fig. 2.The figure shows that the standardized residual value less than three meaning that there are no any influencing cases having the effect in the model.

### Scatter plot for outliers (leverage value)

**Fig. 3:**
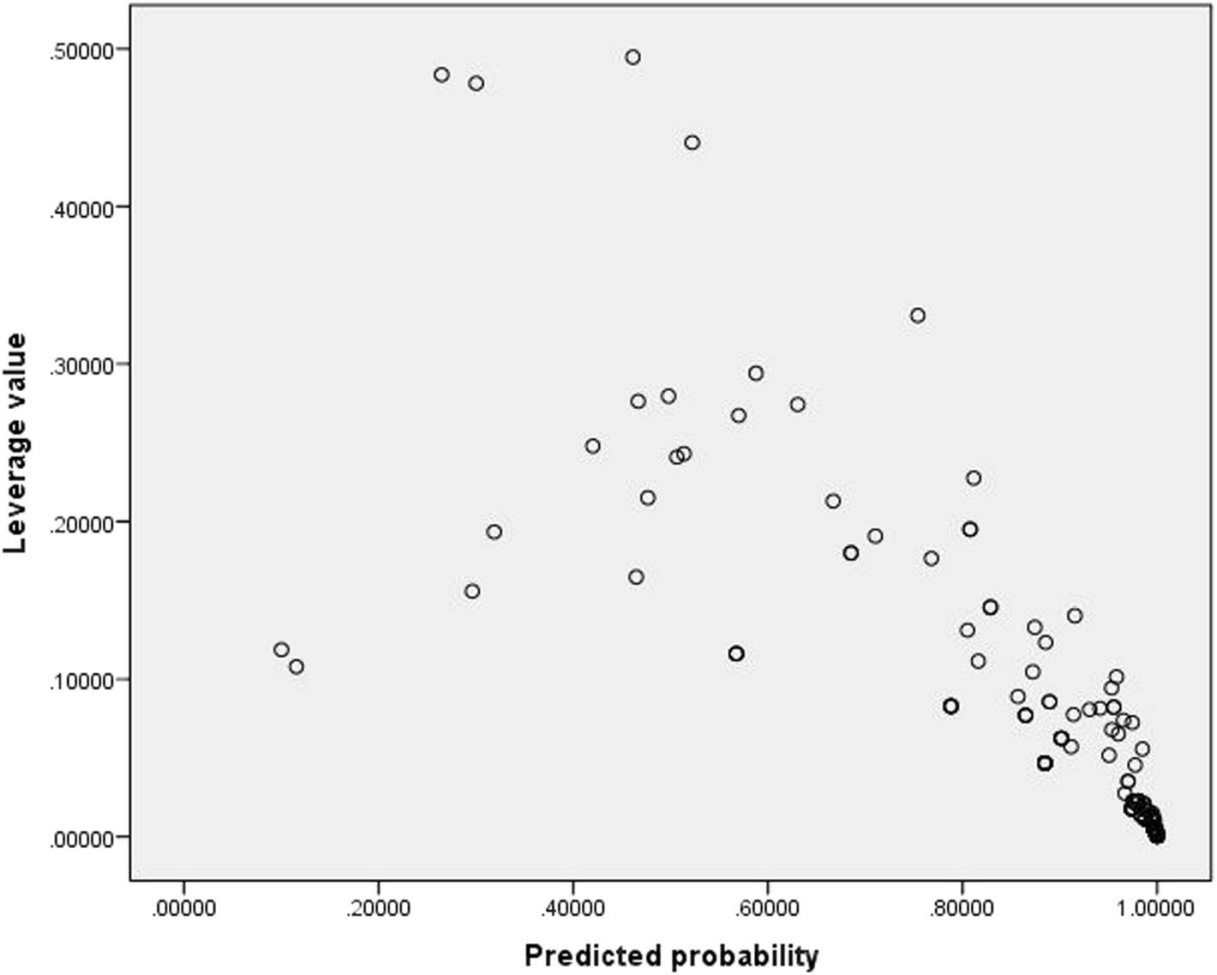
Scatter plot for outliers (Leverage values)

Another method for detecting outliers is leverage value. From the scatter plots of leverage values for the level of adherence as shown in Fig. 3, Leverage values are less than one indicating the absence of outlying observations.

### Scatter plots for outliers (analog of cooks influence)

**Fig. 4:**
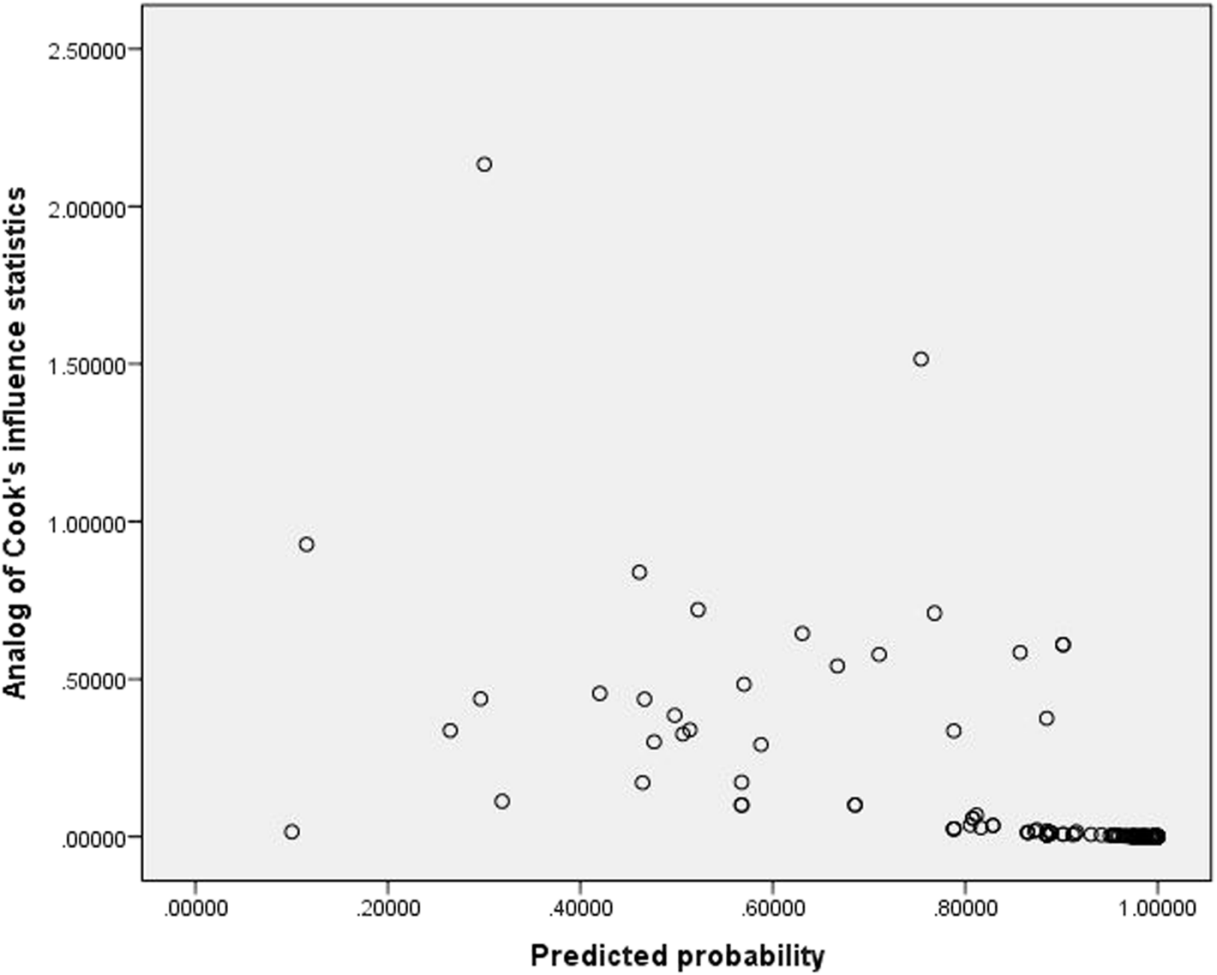
Scatter plot for outliers (Analog of Cooks influence

Cooks distance is proposed to measure the effect of excluding any specific observation on the set of parameter estimates. Cook gives the value of D, d > 1 identifies the case might be influential as shown in Fig. 4 [15].

## Discussion

The present study has attempted to identify the major difficulties faced by ARV users among a representative sample of People Living with HIV and AIDS accessing treatment site in ART centre of Bharatpur, Chitwan, Nepal. The aim of the study was to determine the adherence to Anti-Retroviral Therapy (ART) and its associated factors among People Living with HIV in ART Center of Chitwan, Nepal.

The overall adherence as per the findings of our study was found to be 87.4%. This can be credited to effective mobilization of CHBC team within the community which has resulted in increase awareness level of the community. Even though the adherence rate seemed higher, 12.6% non-adherence observed in present study pose a serious concern in country like Nepal coupled with topographical difficulties and limitation of resources. Non adherence to ART will lead to increase in economic problem for People Living with HIV and AIDS as non adherence will increase in morbidity and mortality and the cost for health care. Having busy schedule and forgetting to take ART were the main reasons for non-adherence.

The finding was successful in addressing the research questions. The adherence rate of the respondents of the ART center of Bharatpur Hospital was 87.4%. The factors associated with adherence among PLHIV in ART center of Bharatpur Hospital were sex, family type, alcohol habit, receiving ART medicine own-self, HIV duration and side effects.

The finding was almost similar to the mixed method study carried out in Nepal by Wasti etal.in 2009 that reported 86% adherence [2]. The findings of our study seem to be consistent with the study carried out in Kathmandu valley by Shigdel et al. in 2012 which reported adherence rate of 86.7% [15].

A cross sectional study which was carried out by Bam et al. in Nepal in 2015 showed the reported adherence of 94.8% which is a bit higher than our findings [7]. The sample size of the study was larger than the sample size of our study which might be the reason for the discrepancy in the adherence rate. Also the study was representative of 12 ART sites of Nepal (Sample size: 231 vs. 435).

A cross sectional study conducted in 116 HIV patients by Achhapa et al. in 2016 in India showed only 64% adherence which is much lower than the findings of our study [16]. The sample size was much lower in comparison to the size of our study that might have been the reason for lower reported adherence rate in India. Similarly the difference was also seen in age group in the study conducted in India and our study. The aforementioned study conducted in India had taken age group above 49 years also which might be the cause for lower adherence as old age people are more likely to have the habit of forgetting the pills.

A cross sectional study conducted in Oromia region, Ethiopia by Yadeta et al. showed the adherence rate of 66.2% which is very low compared to the findings of our study. The region might be the accessibility factor as the aforementioned study reported that distance to health facility was the major reason for non adherence [17].

The variables sex, family type, alcohol habit, Picking up ART medicines on their own, HIV duration and side effects were found to be the strong predictors of ART adherence which is almost similar to mixed method study conducted in Nepal which showed age, alcohol habit, side effects as strong predictor of ART adherence [9].

## Conclusion

This study has identified some of the barriers to adherence in a developing country setting. The adherence 87.4% seems to be encouraging; however achieving adherence for all the patients on ART is a great challenge.

Identifying and evaluating the problems faced by ARV drug users can foster the achievement of ART related goals and addressing ART related problems in a rational way. Effective and appropriate monitoring of non adherence behaviors can help patients increase adherence level fostering improvement in treatment outcome.

In conclusion, for maximizing the benefit of ARV therapy, education on medication adherence for PLHIV is a must. Appropriate social policy and development of supportive environment for PLHIV can be considered beneficial for improvement in adherence rate of PLHIV.

## Additional file

Additional file 1: Semi Structured Questionnaire. (PDF 321 kb)

## Data Availability

The datasets used and/or analyzed during the current study are available from the corresponding author on reasonable request.

## Abbreviations

AACTG: Adult AIDS Clinical Trials Group
AIDS: Acquired immune deficiency syndrome
ART: Antiretroviral therapy
CHBC: Community and Home based care
DCMPH: Department of Community Medicine and Public health
FSWs: Female sex workers
GFATM: Global Fund to Fight AIDS, Tuberculosis and Malaria
HIV: Human immune deficiency virus
HPTN: HIV Prevention and trial network
I NSTIs: Integrase strand transfer Inhibitors
INGO: International Non Governmental Organization
KPs: Key populations
MSM: Men who have sex with men
NGO: Non-Governmental organization
NNRTIs: Non-Nucleoside Reverse Transcriptase Inhibitors
NRTIs: Nucleoside Reverse Transcriptase Inhibitors
NSASC: National centre for AIDS and STD control
NtRTIs: Nucleotide reverse-transcriptase inhibitors
PLHIV: People living with HIV and AIDS
PWID: People who inject drugs
UNAIDS: United Nations Programme on HIV and AIDS
WHO: World Health Organization
