# Supplementary material for: Adherence to antiretroviral treatment and associated factors among people living with HIV and AIDS in CHITWAN, Nepal
Source: BMC Public Health. 2019 Jun 10;19:720. doi: 10.1186/s12889-019-7051-3 (PMC6558692; doi:10.1186/s12889-019-7051-3)
Supplement: Supplementary file 1 — Semi Structured Questionnaire. (PDF 321 kb) [file 12889_2019_7051_MOESM1_ESM.pdf]

APPENDICES

APPENDIX I:

QUESTIONNAIRE SCHEDULE (ENGLISH)

ADHERENCE TO ANITIRETROVIRAL THERAPY AND ITS ASSOCIATED FACTORS AMONG PEOPLE LIVING WITH HIV AND AIDS IN CHITWAN

CONSENT FORM

Namaskar.

My name is Sujan Neupane (Interviewer). I am final year student of Chitwan Medical College studying Masters in Public Health. I am conducting thesis on the topic Adherence to Antiretroviral therapy and associated factors among people living with HIV and AIDS in Chitwan, Nepal. I am conducting this thesis for the partial fulfillment of my master's degree for public health. The purpose of this study is to determine the Adherence level of people living with HIV and AIDS in Chitwan. The following questionnaire has been prepared to study the topic.

I would like to ask you some questions if I may, but you can refuse to answer any questions I ask. You may end the interview at any time. You can also refuse to participate in the study entirely. The interview will be approximately 30-45 minutes. So, I would like to have your cooperation during the study. Please help me by providing the correct answer to each question. All of the information provided by you will be treated as strictly confidential. The information provided by you will be very crucial for my study and for future ART programmes.

May I proceed with the questions?

Yes ………………..

No …..…………..

Name of interviewer: Sujan Neupane

APPENDIX-II

Sample no: …………………..Date: ……………

Risk group: …………………..

Address: Permanent ……… ……… Temporary: ……………………………

S.N	Indicators	Variables	Decode	Skip
**Socio Demographic and economic factors**				
1	Age			
2	Sex	Female<br>Male<br>Third gender	1<br>2<br>3	
3	Ethnicity	Dalit<br>Janajaties<br>Madhesi<br>Muslim<br>Brahmin/ chhetri<br>Others………………	1<br>2<br>3<br>4<br>5<br>9	
4	Religion	Hindu<br>Boudha<br>Islam<br>Christian<br>Others…………………	1<br>2<br>3<br>4<br>9	
5	Marital Status	Married<br>Unmarried<br>Divorced/ Separated<br>Widowed	1<br>2<br>3<br>4	
6	Educational Status of head of family	Illiterate<br>Primary<br>Lower secondary<br>Secondary<br>Higher secondary<br>Graduate or post graduate<br>Profession or honors	1<br>2<br>3<br>4<br>5<br>6<br>7	
7	Type of family	Nuclear<br>Joint	1<br>2	
8	Occupation of head of family			
9	Monthly income of family	.		

**Personal Habits**				
10	How often have you had a drink containing alcohol in the last 30 days?	Never<br>Once a month<br>2-3 times a month<br>Once or twice a week<br>3-4 times a week<br>Nearly every day<br>Daily	0<br>1<br>2<br>3<br>4<br>5<br>6	
11	1How often do you smoke?(last 30 days)	Never<br>1-2 smoke per day<br>3-4 smoke per day<br>5-6 smoke per day<br>More than 6 smoke per day	0<br>1<br>2<br>3<br>4	
12	Heard of HIV and AIDS	Teachers<br>Friends<br>Radio/ Television<br>Newspaper<br>Family members<br>Others	1<br>2<br>3<br>4<br>5<br>9	
13	Heard of ART	Teachers<br>Friends<br>Radio/ Television<br>Newspaper<br>Family members<br>Hospital<br>NGO/INGO<br>Others	1<br>2<br>3<br>4<br>5<br>6<br>7<br>9	
**Knowledge on HIV and AIDS and ART**				
14	What do you know about HIV infection?	➢ HIV is a lifelong infection<br>➢ consistent condom use and having just one uninfected faithful partner can reduce the chances of getting the AIDS virus<br>➢ Healthy-looking person can have the AIDS virus<br>➢ rejecting the two most common local misconceptions about HIV transmission in Nepal: that HIV can be<br>• transmitted by mosquito bites<br>• HIV can be transmitted by sharing food with a person who has AIDS.	1<br>2<br>3<br>4	
15	What do you know about	ART prolongs life	1	

	ART?	ART improves quality of life<br>ART is a lifelong treatment<br>Knowledge about side effects	2<br>3<br>4	
**Duration for HIV infection and ARV Treatment**				
16	Duration of getting HIV infected	Less than 1 year<br>1-3 year<br>More than 3 year	1<br>2<br>3	
17	Duration in ARV treatment	Less than 1 year<br>1-3 year<br>More than 3 year	1<br>2<br>3	

**Disclosure and Stigma**				
18	Disclosure status	Yes<br>No	1<br>0	If no skip to 23
19	If yes to whom have you been disclosed with?	Friends<br>Family members<br>Health workers<br>Others	1<br>2<br>3<br>9	
20	To what extent do your friends or family members help you remember to take your medication?	Not at all<br>A little<br>Somewhat<br>A lot<br>I live alone	0<br>1<br>2<br>3<br>4	
21	In general, how satisfied are you with the overall support you get from your family members?	Very Dissatisfied<br>Somewhat Dissatisfied<br>Somewhat Satisfied<br>Very Satisfied	0<br>1<br>2<br>3	
22	In general, how satisfied are you with the overall support you get from your friends and society?	Very Dissatisfied<br>Somewhat Dissatisfied<br>Somewhat Satisfied<br>Very Satisfied	0<br>1<br>2<br>3	
23	If no then why is the reason for not disclosing?			
24	Have you ever had any experience of being treated differently because of your HIV status?	Yes<br>No	1<br>0	If no skip to 26
25	If yes where?	Family<br>Working place<br>Friends<br>Others …………………	1<br>2<br>3<br>9	
26	Have you ever thought of leaving ART Medication?	Yes<br>No	1<br>0	If no skip to 28

27	If yes then why?			
28	Have you received counseling on ART Adherence?	Yes<br>No	1<br>0	
**How sure you are that (ART Perception)**				
29	You will be able to take all or most of the medication as directed?	Not all sure<br>Somewhat Sure<br>Very Sure<br>Extremely sure	0<br>1<br>2<br>3	
30	The medication will have a positive effect on your health	Not all sure<br>Somewhat Sure<br>Very Sure<br>Extremely sure	0<br>1<br>2<br>3	
31	If you do not take this medication exactly as instructed, the HIV in your body will become resistant to HIV medications	Not all sure<br>Somewhat Sure<br>Very Sure<br>Extremely sure	0<br>1<br>2<br>3	
32	If you do not take this medication exactly as instructed, the viral load increases and patient health will get worse	Not all sure<br>Somewhat Sure<br>Very Sure<br>Extremely sure	0<br>1<br>2<br>3	
33	How would you describe your health since you started treatment?	Better<br>Same<br>Worse	1<br>2<br>3	
**ART Medication questionnaire**				
34	During the last month, have you been prescribed any ART medications?	Yes<br>No	1<br>0	
35	Have you had your medication at right time?	Yes<br>No	1<br>0	
36	Have you skipped any medicine (in the last 30 days)?	Yes<br>No	1<br>0	If no skip to 41
37	When was the last time you missed taking any of your medications?	Within last one week<br>1-2 week before<br>2-4 week before	1<br>2<br>3	
38	How many pills have you missed?			
39	Total number pills that should have been taken?			

**40. We appreciate how difficult it can be to take pills on a daily basis. If you sometimes miss a dose, please can you tell me what causes this to happen? In the past 1 month**				
1	You wanted to avoid side effects?	Never<br>Rarely	0<br>1	

		Sometimes<br>Often	2<br>3	
2	Of sharing anti-HIV medications with other family members and friends?	Never<br>Rarely<br>Sometimes<br>Often	0<br>1<br>2<br>3	
3	Of religious beliefs?	Never<br>Rarely<br>Sometimes<br>Often	0<br>1<br>2<br>3	
4	Of not fully understanding the anti-HIV medications and their requirements?	Never<br>Rarely<br>Sometimes<br>Often	0<br>1<br>2<br>3	
5	Being away from home	Never<br>Rarely<br>Sometimes<br>Often	0<br>1<br>2<br>3	
6	Of transportation problems getting to the clinic?	Never<br>Rarely<br>Sometimes<br>Often	0<br>1<br>2<br>3	
7	Of lost or stolen pills (for example, while in transit in a taxi/bus/train/car)?<br>. …………	Never<br>Rarely<br>Sometimes<br>Often	0<br>1<br>2<br>3	
8	You had too many pills	Never<br>Rarely<br>Sometimes<br>Often	0<br>1<br>2<br>3	
9	You had a bad event happen that you felt was related to taking the pills?	Never<br>Rarely<br>Sometimes<br>Often	0<br>1<br>2<br>3	
10	You forgot?	Never<br>Rarely<br>Sometimes<br>Often	0<br>1<br>2<br>3	
11	You ran out of pills?	Never<br>Rarely<br>Sometimes<br>Often	0<br>1<br>2<br>3	
12	You were busy doing other things (for example, working, trying to survive, getting	Never<br>Rarely	0<br>1	

	food)?	Sometimes<br>Often	2<br>3	
13	Of not having enough food to eat (for example, to take with your pills)?	Never<br>Rarely<br>Sometimes<br>Often	0<br>1<br>2<br>3	
14	Of concern that anti-HIV medications would work so well that you would lose public financial support?	Never<br>Rarely<br>Sometimes<br>Often	0<br>1<br>2<br>3	
15	Of fear of stigmatization or being discriminated against outside the home (for example, what others may say)?	Never<br>Rarely<br>Sometimes<br>Often	0<br>1<br>2<br>3	
16	Of fear of stigmatization or being discriminated against within the home (for example, not wanting husband, wife, partner to know)?	Never<br>Rarely<br>Sometimes<br>Often	0<br>1<br>2<br>3	
17	You felt the anti-HIV medications were toxic or harmful?	Never<br>Rarely<br>Sometimes<br>Often	0<br>1<br>2<br>3	
18	Your pills got damaged by heat or getting wet?	Never<br>Rarely<br>Sometimes<br>Often	0<br>1<br>2<br>3	
19	You were too ill to attend clinic visits to collect medications?	Never<br>Rarely<br>Sometimes<br>Often	0<br>1<br>2<br>3	
20	You felt depressed or overwhelmed?	Never<br>Rarely<br>Sometimes<br>Often	0<br>1<br>2<br>3	
21	You didn’t think they would really work?	Never<br>Rarely<br>Sometimes<br>Often	0<br>1<br>2<br>3	
22	You were bothered by your dreams?	Never<br>Rarely<br>Sometimes<br>Often	0<br>1<br>2<br>3	
23	Other reason? Please specify below			

41	If not, what is it that helps you to take your pills regularly and on time?	Friends<br>Family<br>Mobile<br>Clock<br>Others………………	1<br>2<br>3<br>4<br>9	
42	Are there any other medications you are taking?	Yes<br>No	1<br>2	If no skip to 44
43	If yes, which medications are you using?	Traditional medicine<br>Herbal medicine<br>Medicine from other hospitals<br>Clinic/ Pharmacy<br>Others ……………	1<br>2<br>3<br><br>4<br>99	
44	Have you experienced any side effects with your ART medication?	Yes<br>No	1<br>2	If no skip to 46

45	If yes what are the side effects that you have experienced?	Skin rash<br>Nausea<br>Diarrhea<br>Depression<br>Anemia<br>Fatigue<br>Headache<br>Fever<br>Others……………….	1<br>2<br>3<br>4<br>5<br>6<br>7<br>8<br>9	
46	Do you yourself take medicine regularly from the ART Clinic?	Yes<br>No	1<br>0	If yes skip to 48
47	If not who receives medicine for you?	Friends<br>Family members<br>Health Workers<br>Others	1<br>2<br>3<br>9	
48	At what duration do you come to receive your medicine?	Daily<br>Weekly<br>Monthly<br>Others……………	1<br>2<br>3<br>9	
49	How do you come to this ART center?	By walk<br>By Bus<br>Personal Vehicle<br>Rickshaw/ Cycle	1<br>2<br>3<br>4	

50	How much time does it takes for you to come to this ART center?	30 minutes<br>30 minutes- 1 hour<br>1-2 hour<br>More than 2 hour	1<br>2<br>3<br>4	
51	How much does it costs for you per visit?			
52	Waiting time per visit	15 minutes<br>15 minutes- 1 hour<br>1-2 hour<br>More than 2 hour	1<br>2<br>3<br>4	
53	What do you feel about this waiting time? Is it right?	Yes<br>No	1<br>0	
**Questions on ART Center**<br>**What do you think of the service you receive at this clinic?**				
54	Do you feel listened to?	Yes<br>No	1<br>0	
55	Are you given the chance to state your problems and ask questions?	Yes<br>No	1<br>0	
56	Are you treated with respect?	Yes<br>No	1<br>0	
57	Do you feel you can trust the health workers?	Yes<br>No	1<br>0	
58	Do you have privacy during consultation and counseling?	Yes<br>No	1<br>0	
59	How do you find the environment of the clinic?	Excellent<br>Very good<br>Good<br>Not good	1<br>2<br>3<br>4	
60	Are you satisfied with the facilities you have got?	Yes<br>No	1<br>0	If yes skip to 62
61	If not why?			
62	Do the health workers provide enough information about your disease?	Yes<br>No	1<br>0	
63	If yes what type of information do they provide?			

Thank you for your cooperation
